# Clinical impact of molecular diagnosis in horse allergy

**DOI:** 10.1186/2045-7022-4-S2-P53

**Published:** 2014-03-17

**Authors:** Silvia Uriarte Obando, Joaquín Sastre Domínguez

**Affiliations:** 1 Fundación Jiménez Díaz, Allergy Department, Madrid, Spain

## Background

Currently, it has increased people who practice horse riding, maintaining direct contact with horses. In some cities, horses are within the city as a form of transportation or tourism. Horses have allergens sources, like their dander and saliva. Allergy to horse is a frequent cause of rhinitis, asthma or contact urticaria.The prevalence of sensitization to horse allergens is not well known.

## Methods

We selected 30 patients sensitized to horse. Specific IgE measurement to horse allergens Equ c1 and Equ c3, cat allergen Fel d2 and dog allergen Can f3, was performed by ImmunoCAP® and/or microarray ISAC® (ThermoFisher Scientific, Sweden), a value > 0,35 kU/L or >0,3 ISU was considered as positive, respectively. Associations of Specific IgE measurements and presence and type of rhinitis or asthma were studied. Statistical analysis was performed using Fisher test and chi-squared test.

## Results

70% of patients had specific IgE to Equ c1, and 40% to Equ c3. 60% were monosensitized to Equ c1 and 30% to Equ c3. Only, 10% patients were sensitized to both horse allergens (Equ c1 and Equ c3). Sensitized patients to Equ c3 (horse albumin), 66% were sensitized to Fel d2 (cat albumin) and 58% to Can f3 (dog albumin). Equ c1 was associated with severity of rhinitis (p < 0.001)and Equ c3 with persistence and severity of asthma (p 0.04, p 0.04, respectively), and persistent rhinitis (p 0.01). Sensitization to both horse allergens (Equ c1 and Equ c3) was associated with severity of rhinitis (p < 0.01).

## Conclusion

We show different patterns of sensitization to horse allergens, that are commercially available. we observed high prevalence of sensitization to other animal albumins (up to 66%), as cross-reactivity proteins. Severity and persistence both of rhinitis as asthma seems to be associated to the sensitization to some horse allergen.

